# Household

**Published:** 1887-07

**Authors:** 


### HOUSEHOLD.

PICTURES—A room with pictures in it, and a room without pictures, differ by nearly as much as a room with windows and a room without windows. Nothing, we think, is more melancholy, particularly to a person who has to pass much time in his room, than blank walls and nothing on them; for pictures are loopholes of escape to the soul, leading it to other scenes and other spheres. It is such an inexpressible relief to a person engaged in writing, or even reading, on looking up, to find his soul escaping, as it were, through the frame of an exquisite picture, to other beautiful, and perhaps idyllic scenes, where fancy for a moment may revel, refreshed and delighted. Is it winter in your world? Perhaps it is summer in the picture. What a charming momentary change and contrast! And thus pictures are consolers of loneliness; they are a sweet flattery to the soul; they are a relief to the jaded mind; they are windows to the imprisoned thought; they are books; they are histories and sermons, which we can read without the trouble of turning over the leaves.—*The Earth.*

HOUSEHOLD HINTS.—Boiling in strong soapsuds will clean up an old lampburner and make it as good as new.

Oil-cloths can be brightened, after washing, by rubbing hard with a flannel moistened with kerosene.

Any gold jewelry that an immersion in water will not injure, can be beautifully cleaned by shaking it well in a bottle nearly half full of warm soapsuds, to which a little prepared chalk has been added, and after rinsing in clear, cold water and wiping it dry.

Take equal parts of flour, grated cheese and butter; season with pepper and salt, and mix with one or two eggs. Bake in small cake or patty pans. You will call them nice cheese cakes.

MINCED VEAL.—Cut the meat from the bones, and having minced it very fine with a small piece of lemon peel, grate over it a little nutmeg, and sprinkle on some pepper and salt. Put into a saucepan with a large onion chopped fine, and water enough to moisten well; thicken with a little flour and butter and serve on buttered toast.—*The Household.*

Cheap Cake.—Two cups of molasses, one and a half cups of boiling water, two tablespoonsful (not heaped) of tried-out beef suet, or a piece of butter the size of an egg, three heaped tablespoonsful of baking powder, spices to taste, and a cup of raisins and currants. Add flour enough to make a soft batter, and bake in loaves.

Puff Pudding.—One pint of boiling milk and nine tablespoonsful of flour, mixed first with a little cold milk. When cold, add a little salt and four well-beaten eggs and bake in a buttered dish. Serve as soon as it is done.

Muffins in Tins.—Take one cup of sour milk, one egg, a little shortening, a teaspoonful of bicarbonate of soda ; if the milk is not very sour less soda will do. Make a thick batter, add a little salt, and bake in a hot oven. If you cannot obtain sour milk, sweet milk and baking powder will answer. To a teacup of sweet milk allow a heaping teaspoonful of baking powder.

Cornmeal Short Cake.—Two cups of Indian meal and one of flour, sifted into a bowl with a teaspoonful of soda and the same of salt ; sift three times : one tablespoonful of butter and two of lard, two tablespoonfuls of sugar, three eggs, two cups of "loppered" milk. Rub sugar and shortening together, beat the eggs light and then add the milk, lastly the mingled flour and meal ; bake in a square, shallow pan, and when done cut in squares. Split and eat hot.

Parker House Rolls.—One pint of cold boiled milk, one teaspoonful of salt, two quarts of sifted flour, one large spoonful of lard, one teaspoonful of sugar, one-half cup of yeast or half a compressed cake, dissolved in a half cup of lukewarm water. Put the flour in a deep bowl, add salt and sugar. Mix and then rub in the lard. Make a hole in centre. Mix the yeast and milk well together, pour it into the flour and let it stand until morning. Then stir and knead thoroughly, first in the bowl, and, as soon as stiff enough, on the board. Now pound for fifteen minutes with a potato masher ; as soon as it becomes velvety put it back in the bowl, cover it, and set away in a warm place until very light. Roll out on the board in a paste a quarter of an inch thick, cut with a round cutter. Mrs. Rorer says, you must remember that different kinds of flour require more or less moisture. Do not add the whole two quarts if less will serve.

When you wish to boil cream or milk in a saucepan, do not put butter in first, as it is apt to scorch and disfigure the cream, but simply rinse out the pan with hot water, and the milk will not stick and burn if ordinary care is used. The cream should be ready boiled and salted before the bread is toasted, as it is important to dip the bread before it toughens and cools. If milk is used, it is better to be *very slightly* thickened with flour. Unless the bread is quite stale it should be dried before toasting. Usually baker's bread makes better toast than home-made bread.

